# Intracellular β-glucosidase regulates cellulase expression and development in *Aspergillus nidulans*

**DOI:** 10.1007/s00253-026-13851-9

**Published:** 2026-05-04

**Authors:** Shun Yakabe, Chihiro Kadooka, Tomohiko Matsuzawa, Yuzuki Kawai, Masayuki Noguchi, Daisuke Hira, Masatoshi Goto, Takuji Oka

**Affiliations:** 1 Division of Applied Microbial Technology, Graduate School of Engineering, Sojo University, 4-22-1 Ikeda, Nishi-Ku, Kumamoto, 860-0082 Japan; 2 Department of Biotechnology and Life Sciences, Faculty of Biotechnology and Life Sciences, Sojo University, 4-22-1 Ikeda, Nishi-Ku, Kumamoto, 860-0082 Japan; 3 Department of Applied Biological Science, Faculty of Agriculture, Kagawa University, 2393 Ikenobe, Miki, Kagawa 761-0795 Japan; 4 Faculty of Agriculture, Saga University, 1 Honjo, Saga, 840-8502 Japan

**Keywords:** *Aspergillus nidulans*, β-Glucosidase, Glycoside hydrolase family 1, Cellulase regulation

## Abstract

**Abstract:**

β-Glucosidases (EC 3.2.1.21) are essential enzymes involved in biomass degradation and metabolic regulation, but the physiological roles of intracellular β-glucosidases in filamentous fungi remain incompletely understood. In this study, we characterized CbgA (AN10124) and CbgB (AN10375), two intracellular glycoside hydrolase family 1 β-glucosidases, in *Aspergillus nidulans*. Gene deletion and biochemical analyses demonstrated that CbgA is the predominant intracellular β-glucosidase. Loss of *cbgA* led to overactivation of cellulases, cellobiose-dependent accumulation of reddish-brown secondary metabolites, and a significant reduction in conidiation. Crucially, deletion of the cellobiose transporter gene *cltB* in the Δ*cbgA* background markedly attenuated these phenotypes, providing direct genetic evidence that the Δ*cbgA*-associated defects are driven by intracellular cellobiose accumulation rather than energy deficiency. Our findings identify CbgA as a critical “signal gatekeeper” that modulates the intensity of cellobiose-dependent induction. By maintaining the intracellular cellobiose pool within a physiological range, CbgA prevents secretory overload and maintains the metabolic balance between primary development and secondary metabolism. This study clarifies the coordination between nutrient transport and intracellular metabolism in shaping global regulatory outputs, suggesting that the targeted modulation of CbgA activity represents a potential strategy for optimizing cellulase production in fungal cell factories.

**Key points:**

- *CbgA negatively regulates cellulase expression by controlling intracellular cellobiose levels.*
- *Loss of cbgA leads to hyperpigmentation and reduced conidiation on cellobiose.*
- *Deletion of cltB alleviates ΔcbgA phenotypes, confirming the role of intracellular cellobiose.*

**Supplementary Information:**

The online version contains supplementary material available at 10.1007/s00253-026-13851-9.

## Introduction

β-Glucosidases (EC 3.2.1.21) comprise a class of glycoside hydrolases (GHs) that hydrolyze β-glucosidic bonds in carbohydrates and glycosides (Ketudat Cairns and Esen 2010). These enzymes are widely distributed across *Archaea*, *Bacteria*, and *Eukarya*, and they control diverse and often essential functions in each biological context (Morant et al. 2008; Singh et al. 2016; Erkanli et al. 2024; Stathaki et al. 2024; Yang et al. 2024; Nimker et al. 2025). In microorganisms, β-glucosidases are key enzymes in biomass degradation, facilitating the conversion of lignocellulosic materials into fermentable sugars (Yang et al. 2024). In animals, these enzymes help catabolize glycosphingolipids and detoxify exogenous glucosides (Morant et al. 2008; Ketudat Cairns and Esen 2010).

A critical biotechnological function of β-glucosidases is their role in the complete saccharification of cellulose. Cellulose, a major structural component of plant cell walls, is composed of linear β−1,4–linked glucose units (Somerville 2006). Enzymatic degradation of cellulose typically involves the synergistic action of three enzyme classes: endoglucanases, which cleave internal bonds within cellulose chains; cellobiohydrolases, which release cellobiose units from chain ends; and β-glucosidases, which hydrolyze cellobiose into glucose (Lynd et al. 2002). In addition to these hydrolytic enzymes, lytic polysaccharide monooxygenases (LPMOs) play a crucial role by oxidatively cleaving the recalcitrant crystalline regions of cellulose, acting synergistically to significantly enhance degradation (Contato et al. 2025; Forsberg et al. 2019). The final hydrolysis step by β-glucosidases is critical, as cellobiose accumulation both limits the efficiency of cellulose degradation because of product inhibition of upstream cellulases and serves as a regulatory signal influencing cellulase gene expression in many fungi (Lynd et al. 2002; Znameroski et al. 2012). Consequently, β-glucosidases are widely applied in agricultural, environmental, and industrial processes, including biofuel production from lignocellulosic biomass (Kannan et al. 2023).

Given their functional diversity, β-glucosidases have been extensively classified according to their sequence similarity and structural features. The Carbohydrate-Active enZYmes (CAZy) database organizes carbohydrate-active enzymes into families based on conserved sequence motifs and overall amino acid similarity (Drula et al. 2022; Lombard et al. 2014). To date, CAZy lists 194 glycoside hydrolase (GH) families, with β-glucosidases represented mainly in GH1, GH2, GH3, GH5, GH30, GH39, GH116, GH131, GH175, and GH180 (Drula et al. 2022; Lombard et al. 2014), and most β-glucosidases belong to GH1 and GH3. In particular, GH1 β-glucosidases are notable for their broad substrate specificity and structural versatility, which underpin their functional diversity across taxa (Ketudat Cairns and Esen 2010).

In filamentous fungi, considerable research has focused on extracellular β-glucosidases that directly participate in cellulose degradation (Barnett et al. 1991; Kawaguchi et al. 1996). These enzymes are typically secreted, and they coordinate with other cellulolytic enzymes to depolymerize plant biomass in the extracellular environment. However, certain β-glucosidases lack N-terminal signal peptides for secretion, resulting in their cytoplasmic localization (Ketudat Cairns and Esen 2010). Despite the prevalence of intracellular β-glucosidases, their physiological functions in fungi remain poorly characterized (Ketudat Cairns and Esen 2010). Recent studies in filamentous fungi have suggested that intracellular β-glucosidases can influence cellulase induction, based mainly on phenotypes observed after gene deletion. In *Trichoderma reesei*, the intracellular GH1 β-glucosidases Cel1A and Cel1B were found to contribute to efficient cellulase induction, and their roles have been discussed in connection with the formation and turnover of β-linked oligosaccharides (e.g., sophorose) rather than simple hydrolysis of cellobiose (Pang et al. 2021). Related ideas have also been postulated in *Penicillium*, in which intracellular β-glucosidase activity has been linked to the formation of putative inducing disaccharides such as gentiobiose. However, the identities of the key metabolites and the intracellular steps that connect β-glucosidase activity to transcriptional outputs remain incompletely defined across systems (Chen et al. 2013). More recently, in *Aspergillus niger*, the intracellular β-glucosidase Bgl1B was reported to affect the gene expression of lignocellulose-degrading enzymes in both deletion and overexpression strains and generate sophorose and laminaribiose via transglycosylation (Zhang et al. 2025). Although these studies collectively support the idea that intracellular β-glucosidases can reshape cellulase induction profiles, direct causal evidence that specific changes in intracellular pools of cellobiose or related disaccharides drive transcriptional regulation remains limited. Thus, the relevant metabolites, cellular compartments, and transport processes that couple intracellular β-glucosidase activity to induction remain to be fully resolved. In this context, genetic evidence linking uptake processes to intracellular β-glucosidase–associated phenotypes can be particularly informative for defining the point of connection among sugar transport, intracellular metabolism, and cellulase induction.

*Aspergillus* species are ecologically ubiquitous, and they carry considerable industrial, agricultural, and medical importance. Among them, *Aspergillus nidulans* is a valuable experimental model owing to its well-established genetic tools, well-annotated genome, and tractable sexual cycle (Galagan et al. 2005). Because cellobiose-dependent responses in filamentous fungi involve both uptake and intracellular processing, transporter function is an important component of the regulatory machinery. In this context, the present study investigated *cbgA* (AN10124) and *cbgB* (AN10375), two predicted intracellular GH1 β-glucosidases of *A. nidulans*, together with the contribution of cellobiose transport to the observed phenotypes. We characterized their biochemical properties, assessed their relative contributions to intracellular β-glucosidase activity, determined their subcellular localization patterns, and evaluated their roles in regulating cellulase production, including genetic tests that link cellobiose transporter-dependent uptake to *cbgA*-associated phenotypes.

## Materials and methods

### Strains and culture conditions

*Escherichia coli* DH5α (Toyobo, Osaka, Japan) was used for plasmid propagation. For recombinant protein expression, SHuffle T7 Express (New England Biolabs, Ipswich, MA, USA) carrying the pRARE plasmid (MilliporeSigma, Burlington, MA, USA) was used for CbgA and CbgB (Kadooka et al. 2022b). The *A. nidulans* strains AKU89 (*biA1*, ornithine carbamoyl transferase [*argB2*], Δ*nkuB::aurA*^+^), AKU89P (*biA1*, *argB2*, Δ*nkuB::aurA*^+^, Δ*pyrG*), and AKU89A (*biA1*, *argB2::argB*, Δ*nkuB::aurA*^+^) served as the parental strains for gene deletion and phenotypic analyses (Supplementary Table S1) (Goto et al. 2009; Kadooka et al. 2023; Kadooka and Oka 2024). *E. coli* strains were cultivated in Luria–Bertani (LB) medium at 37 °C with appropriate antibiotics, specifically ampicillin (50 µg/mL), chloramphenicol (34 µg/mL), and spectinomycin (25 µg/mL) as required. *A. nidulans* strains were grown on minimal medium (MM) containing 0.6% NaNO_3_, 0.052% KCl, 0.052% MgSO_4_, 0.152% KH_2_PO_4_, and Hutner’s trace elements and supplemented with biotin (0.02 µg/mL) and other nutrients according to auxotrophic requirements (e.g., 1 g/L arginine, 0.1 g/L uridine, 1 g/L uracil) (Barratt et al. 1965). Regarding carbon sources, 1% (w/v) cellobiose or glucose was used unless otherwise specified. For protoplast transformations, MM was supplemented with 0.6 M KCl as an osmotic stabilizer.

### Plasmid construction for recombinant protein expression

The *cbgA* (AN10124) coding sequence was commercially synthesized (Eurofins Genomics, Ebersberg, Germany; Supplementary Table S2). *cbgB* (AN10375) cDNA was obtained by fusion RT-PCR from the total RNA of *A. nidulans*. Total RNA extraction and first-strand cDNA synthesis were performed as described previously (Oka et al. 2015), and the *cbgB* coding region was amplified using the primers pET15-SmaI-CbgB-F and pET15-SmaI-CbgB-R (Supplementary Table S2). PCR products were cloned into the pET15-SmaI vector (Kadooka et al. 2024) at the *Sma*I site using the In-Fusion Cloning Kit (Takara Bio Inc., Kusatsu, Japan), yielding pET15-SmaI-CbgA and pET15-SmaI-CbgB. All constructs were verified by DNA sequencing and propagated in *E. coli* DH5α. Primer sequences are listed in Supplementary Table S2.

### Recombinant protein expression and purification

For recombinant protein production, *E. coli* cells harboring expression plasmids were grown overnight at 37 °C in LB medium with appropriate antibiotics. Cultures were diluted into fresh LB to an OD_600_ of approximately 0.2, induced with 1 mM isopropyl-β-D-thiogalactopyranoside (IPTG), and incubated at 18 °C for 65 h, as described previously (Katafuchi et al. 2017). Cells were harvested (6000 × *g*, 10 min, 4 °C); resuspended in 50 mM 4-(2-hydroxyethyl)−1-piperazineethanesulfonic acid (HEPES) (pH 6.8) containing 100 mM NaCl, 30 mM KCl, and 5% glycerol; and lysed via sonication on ice. After centrifugation (12,000 × *g*, 30 min, 4 °C), the soluble fraction was applied to Ni–NTA agarose (FUJIFILM Wako Pure Chemicals, Osaka, Japan), washed with buffer containing 20 mM imidazole, and eluted with 300 mM imidazole. Purity was assessed by SDS–PAGE with Coomassie Brilliant Blue R-250 staining. Protein concentrations were determined using the Qubit Protein Assay Kit (Thermo Fisher Scientific, Waltham, MA, USA). Typical yields were 1.27 and 1.13 mg/L for CbgA and CbgB, respectively.

### Enzyme activity assays

Enzymatic activity was evaluated using *p*-nitrophenyl (pNP) glycosides and disaccharide substrates. For pNP assays, the standard reaction mixture (20 µL) contained 5 mM pNP substrate, 5 µL of buffer (McIlvaine buffer for pH 5.0–8.0; or 200 mM sodium phosphate buffer for temperature optimization), and 4 µg of enzyme. Mixtures were incubated at specified temperatures (25 °C–45 °C), and reactions were stopped by adding 100 µL of 1 M NaHCO_3_. Absorbance was measured at 405 nm. One unit of enzyme activity was defined as the amount of enzyme that released 1 µmol of pNP per minute under the assay conditions. Substrate specificity was tested against 16 *p*NP glycosides (*p*NP-α-**l**-arabinofuranoside, *p*NP-α-**l**-arabinopyranoside, *p*NP-β-**l**-arabinopyranoside, *p*NP-β-**d**-cellobioside, *p*NP-α-**l**-fucopyranoside, *p*NP-β-**d**-fucopyranoside, *p*NP-β-**l**-fucopyranoside, *p*NP-α-**d**-galactopyranoside, *p*NP-β-**d**-galactopyranoside, *p*NP-α-**d**-glucopyranoside, *p*NP-β-**d**-glucopyranoside, *p*NP-α-**d**-mannopyranoside, *p*NP-β-**d**-mannopyranoside, *p*NP-α-**l**-rhamnopyranoside, *p*NP-α-**d**-xylopyranoside, and *p*NP-β-**d**-xylopyranoside). All *p*NP glycosides were purchased from Sigma-Aldrich (St. Louis, MO, USA), except for *p*NP-α-**d**-glucopyranoside and *p*NP-β-**d**-xylopyranoside (Nacalai Tesque, Kyoto, Japan), and *p*NP-α-**d**-mannopyranoside (FUJIFILM Wako Pure Chemicals, Osaka, Japan). For the activity assays using β-linked glucobioses (cellobiose, sophorose, laminaribiose, and gentiobiose), the reaction mixture (20 µL) contained 2 mM glucobiose substrate, 5 mM HEPES buffer (pH 6.8), and 4 µg of enzyme solution. The reaction was conducted at 40 °C for 5 min and terminated via heating at 99 °C for 5 min. The reaction products were derivatized with 4-aminobenzoic acid ethyl ester (Yasuno et al. 1997) and analyzed by HPLC using an NH2P-50 4E column (Resonac Corporation, Tokyo, Japan) with fluorescence detection (excitation, 305 nm; emission, 360 nm) (Kadooka et al. 2022a, b). Glucose tolerance was assessed by measuring enzyme activity with *p*NP-β-**d**-glucopyranoside in the presence of glucose (0–1000 mM). Solvent resistance was determined by measuring activity in the presence of various concentrations (0–30% v/v) of ethanol, methanol, or dimethyl sulfoxide (DMSO).

### Construction of the pHSG396-*A. nidulans argB* plasmid

*argB* was amplified by PCR using *A. nidulans* A26 genomic DNA as the template and pHSG396-argB-IF-F and pHSG396-argB-IF-R as the primers (Supplementary Table S2). The amplified fragment was inserted into the *Bam*HI site of pHSG396 using the In-Fusion HD Cloning Kit (Takara Bio Inc.) to yield pHSG396-*argB*.

### Construction of single- and double-deletion mutants of* CbgA *and* CbgB *in* A. nidulans*

Single-gene deletion mutants of *CbgA* and *CbgB* were generated using *A. nidulans* AKU89 as the parental strain. In both single-deletion mutants (Δ*cbgA* and Δ*cbgB*), *argB* was used as the selection marker. For each target gene, approximately 1-kb 5′ and 3′ flanking regions were amplified using the primer sets cbgA-1/cbgA-2 and cbgA-3/cbgA-4 or cbgB-1/cbgB-2 and cbgB-3/cbgB-4 (Supplementary Table S2), respectively. The *argB* marker fragment was amplified by pHSG396-F and pHSG396-R using pHSG396-argB as the template (Supplementary Table S2). The 5′ flank, marker, and 3′ flank fragments were fused by fusion PCR, and the full-length replacement cassette was amplified using cbgA-1/cbgA-4 or cbgB-1/cbgB-4 (Supplementary Table S2). Protoplast transformation was then performed, and transformants were selected on MM without arginine supplementation. Correct gene replacement was confirmed by diagnostic PCR using cbgA-FC/cbgA-RC or cbgB-FC/cbgB-RC (Supplementary Table S2).

*A. nidulans* AKU89P was used as the parental strain to construct the Δ*cbgA*Δ*cbgB* double mutant. First, *cbgB* was deleted using *pyrG* as the marker. The *pyrG* marker fragment was amplified by pHSG396-F and pHSG396-R using pHSG396-*AnpyrG* (Kadooka et al. 2022b) as the template. The *cbgB* replacement cassette was assembled by fusion PCR using the *cbgB* flanking fragments (cbgB-1/cbgB-2 and cbgB-3/cbgB-4) and *pyrG* marker fragment, and the full-length cassette was amplified by cbgB-1/cbgB-4 (Supplementary Table S2). Protoplast transformation was performed, and transformants were selected on MM supplemented with arginine (1 g/L), without uridine and uracil supplementation. The resulting Δ*cbgB* strain (*argB2*) was then used as the recipient for *cbgA* deletion using *argB*. The *argB* marker fragment was amplified by pHSG396-F and pHSG396-R using pHSG396-*argB* as the template (Supplementary Table S2). The *cbgA* replacement cassette was assembled by fusion PCR using the *cbgA* flanking fragments (cbgA-1/cbgA-2 and cbgA-3/cbgA-4) and *argB* marker fragment, followed by amplification with cbgA-1/cbgA-4 (Supplementary Table S2). Transformants were selected on MM. Correct gene replacement at each locus was confirmed by diagnostic PCR using cbgA-FC/cbgA-RC or cbgB-FC/cbgB-RC (Supplementary Table S2).

### Construction of Δ*cbgA*Δ*cltA* and Δ*cbgA*Δ*cltB* double mutants

To examine the dependence of Δ*cbgA*-associated phenotypes on cellobiose uptake, double mutants lacking *cbgA* and a cellobiose transporter gene (*cltA* or *cltB*) were constructed using *A. nidulans* AKU89P as the parental strain. First, *cbgA* was deleted using *argB* to generate the Δ*cbgA* (∆*pyrG*) strain. The *cbgA* replacement cassette was assembled by fusion PCR using the *cbgA* flanking fragments (cbgA-1/cbgA-2 and cbgA-3/cbgA-4) and the *argB* marker fragment amplified with pHSG396-F and pHSG396-R from pHSG396-*argB*, and the full-length cassette was amplified using cbgA-1/cbgA-4 (Supplementary Table S2). Transformants were selected on MM supplemented with uridine (0.1 g/L) and uracil (1 g/L). Correct gene replacement was confirmed by diagnostic PCR using cbgA-FC/cbgA-RC.

The Δ*cbgA* strain (Δ*pyrG*) was subsequently used as the recipient for *cltA* or *cltB* deletion using *pyrG*. For *cltA* and *cltB* deletion, approximately 1-kb 5′ and 3′ flanking regions were amplified using the primer sets cltA-1/cltA-2 and cltA-3/cltA-4 or cltB-1/cltB-2 and cltB-3/cltB-4, respectively (Supplementary Table S2). The *pyrG* marker fragment was amplified by pHSG396-F and pHSG396-R using pHSG396-*AnpyrG* as the template (Supplementary Table S2). The *cltA* or *cltB* replacement cassette was assembled by fusion PCR and amplified using cltA-1/cltA-4 or cltB-1/cltB-4 (Supplementary Table S2). Protoplast transformation was performed using the Δ*cbgA* strain as the recipient, and transformants were selected on MM. Correct gene replacement at each locus was verified by diagnostic PCR using cltA-FC/cltA-RC or cltB-FC/cltB-RC (Supplementary Table S2).

### Colony phenotypic analysis

For phenotypic analysis, conidial suspensions (1 × 10^4^ conidia in 1 µL) were spotted onto MM plates containing either glucose or cellobiose as the sole carbon source and incubated at 37 °C for 3 days.

### Construction of the *cbgA* complementation strain

For complementation analysis, a genomic DNA fragment containing the *cbgA* coding region along with its native promoter and terminator regions was amplified by PCR using AKU89 genomic DNA as the template with the primers pPTR-II-cbgA-F and pPTR-II-cbgA-R (Supplementary Table S2). The resulting PCR product was inserted into the *Sma*I site of the autonomously replicating plasmid pPTR-II (Takara Bio Inc.) using the In-Fusion HD Cloning Kit (Takara Bio Inc.), yielding the complementation plasmid pPTR-II-cbgA. The plasmid was introduced into the strain via the protoplast-PEG method. Transformants were selected on MM supplemented with 0.1 g/mL pyrithiamine.

### Intracellular β-glucosidase activity

To quantify intracellular β-glucosidase activity, cytosolic fractions were extracted from mycelia, and β-glucosidase activity was determined to evaluate the contributions of CbgA and CbgB in *A. nidulans*. The AKU89A, Δ*cbgA*, Δ*cbgB*, and Δ*cbgA*Δ*cbgB* strains were cultured in liquid MM supplemented with 1% (w/v) cellobiose at 37 °C for 24 h with shaking. Mycelia were collected via filtration through gauze and suspended in a maleate buffer solution comprising 0.6 M (NH_4_)_2_SO_4_ and 50 mM maleate buffer (pH 5.5), supplemented with 0.1 g/mL VinoTaste Pro (Novozymes, Bagsvaerd, Denmark), and incubated at 30 °C for 3 h to generate protoplasts. The resulting protoplast suspension was filtered through Miracloth (MilliporeSigma, Burlington, MA, USA) to remove debris, and the filtrate was centrifuged at 2000 × *g* for 5 min to collect the protoplasts. The harvested protoplasts were then washed three times with McIlvaine buffer (pH 7.0) containing 0.6 M KCl. Protoplasts were then pelleted by centrifugation (2000 × *g*, 5 min, 4 °C) and lysed in McIlvaine buffer (pH 7.0) without 0.6 M KCl to release the cytosolic fraction. The lysate was centrifuged at 20,000 × *g* for 5 min at 4 °C, and the supernatant was collected and used as the enzyme solution. β-Glucosidase activity was measured using *p*NP-β-**d**-glucopyranoside as the substrate. The reaction products were quantified by measuring the absorbance of the released pNP at 405 nm using a microplate reader (Multiskan FC Microplate Photometer, Thermo Fisher Scientific, Waltham, MA, USA).

### Construction of eGFP fusion protein expression plasmids and subcellular localization of *CbgA* and *CbgB*

To generate DNA constructs for eGFP fusion proteins, regions spanning from approximately 1000 bp upstream of the start codon to immediately before the stop codon of each target gene were amplified by PCR. Genomic DNA was used as a template using AN8041-F and AN8041-R for *gpdA*, AN10124-F and AN10124-R for *cbgA*, and AN10375-F and AN10375-R for *cbgB* (Supplementary Table S2). Separately, eGFP, incorporating a linker sequence, was amplified using the pEGFP plasmid (Takara Bio Inc.) as a template with the primers (GGGGS)_2_-eGFP-F and (GGGGS)_2_-eGFP-R (Supplementary Table S2). Subsequently, fusion PCR was performed using the amplified products of each gene and the amplified (GGGGS)_2_-tagged eGFP fragment as the template. Regarding the primer combinations for fusion PCR, AN10124-F, AN10375-F, or AN8041-F was used together with the (GGGGS)_2_-eGFP-R primer (Supplementary Table S2). The resulting fusion PCR products were then cloned into the *Sma*I site of the autonomous replicating plasmid pPTR-II using the In-Fusion Cloning Kit (Takara Bio Inc.), thereby generating the expression plasmids pPTR-II-GpdA-eGFP, pPTR-II-CbgA-eGFP, and pPTR-II-CbgB-eGFP. The constructed plasmids were used to transform *A. nidulans* via the protoplast-PEG method, and localization of the expressed eGFP fusion enzymes was subsequently confirmed using fluorescence microscopy (BZ-X800, KEYENCE, Osaka, Japan).

### Zymogram analysis of cellulase production

To evaluate broader extracellular cellulase activities, primarily endoglucanases, we used carboxymethyl cellulose (CMC) as a polymeric substrate because it more closely resembles natural cellulose than *p*NP substrates. Cellulase activity in culture supernatants was detected using a zymogram-based assay, which provides direct visual confirmation of the secretion levels. *A. nidulans* strains were grown in liquid MM supplemented with either 1% (w/v) cellobiose alone or together with 1% (w/v) glucose as the carbon source for 24 h at 37 °C with shaking. After cultivation, 50 mL of culture supernatant was collected and concentrated to 1 mL using an Amicon® Ultra Centrifugal filter (MilliporeSigma, Burlington, MA, USA). Native polyacrylamide gels (10%) were prepared according to the standard protocol, and the concentrated samples were loaded onto the gels. Electrophoresis was performed at a constant current of 18 mA for 90 min. Following electrophoresis, the gel was placed in a tray, overlaid with 1% (w/v) carboxymethyl cellulose solution, and incubated at 50 °C for 1 h to permit enzymatic hydrolysis. The gel was then stained with 0.1% (w/v) Congo red for 10 min, followed by destaining with 1 M NaCl until clear cellulase activity bands were visible.

## Results

### Selection of intracellular β-glucosidases

According to the CAZy database, the *A. nidulans* genome contains four putative β-glucosidases belonging to the GH1 family: AN9183, AN10124, AN10353, and AN10375. One of these is AN10353, which has been previously reported as SgdB (Osherov and May 2000), a protein involved in spore germination with homologies to seryl-tRNA synthetase and β-glucosidase. To determine the target genes for this study, we predicted the subcellular localizations of these four GH1 proteins using DeepLoc 2.1 (Ødum et al. 2024). The analysis predicted that AN10124 and AN10375 localize to the cytoplasm, whereas AN9183 and AN10353 were predicted to be localized to the extracellular space and mitochondrion, respectively. Furthermore, SignalP 6.0 analysis revealed the presence of a secretory signal peptide only in AN9183, while no such sequence was found in the other three proteins (Teufel et al. 2022). Additionally, amino acid sequence alignment of these four GH1 proteins revealed that AN10124 and AN10375 share a sequence identity of 51.3%. In contrast, their sequence identities with AN9183 were notably lower (20.2% and 20.5%, respectively), and both shared less than 10% identity with AN10353. Due to this remarkably low homology, AN10353 was excluded from the multiple sequence alignment (Thompson et al. 1994; Supplementary Fig. S1). Importantly, the alignment demonstrated that both AN10124 and AN10375 perfectly conserve the acidic catalytic residues typical of GH1 family enzymes, corresponding to E173 and E384 in AN10124 (Erkanli et al. 2024; Supplementary Fig. S1). Based on these in silico predictions and sequence similarities, we hypothesized that AN10124 and AN10375 are functional β-glucosidases localized in the cytosol. Consequently, we designated them as cytosolic β-glucosidase A (*cbgA*) and B (*cbgB*) and focused on these two genes for further characterization.

### Enzymatic properties of CbgA and CbgB

Recombinant CbgA and CbgB, each containing an N-terminal 6×His tag, were successfully expressed in *E. coli* and purified by Ni–NTA affinity chromatography. SDS–PAGE revealed a single major band at approximately 55 kDa for both enzymes (Supplementary Fig. S2A), consistent with the predicted molecular mass of 54.9 kDa for CbgA and 55.5 kDa for CbgB. To survey substrate specificity, we first assessed enzymatic activity using 16 different pNP-glycoside substrates (Supplementary Fig. S2B). Based on this screening, *p*NP-β-**d**-glucopyranoside, *p*NP-β-**d**-galactopyranoside, and *p*NP-β-**d**-fucopyranoside were selected for subsequent kinetic characterization. The steady-state kinetic parameters for these representative substrates are summarized in Table 1. CbgA exhibited exceptionally high catalytic efficiency toward *p*NP-β-**d**-glucopyranoside, a glucose-releasing substrate and a proxy for β-glucosidic bond hydrolysis (*k*_cat_/* K*_m_ = 2.90/s·mM), which was more than 2.5-fold higher than that of CbgB (1.13/s·mM; Table 1). For *p*NP-β-**d**-fucopyranoside, CbgA also exhibited higher catalytic efficiency (*k*_cat_/*K*_m_ = 42.8/s·mM) than CbgB (34.5/s·mM; Table 1). This difference was also evident in kinetic analyses using *p*NP-β-**d**-galactopyranoside, which revealed distinct catalytic efficiencies (Table 1).

**Table 1:** Kinetic parameters of recombinant CbgA and CbgB toward various *p*-nitrophenyl (*p*NP) glycoside substrates

Substrate	Enzyme	*K*_m_ (mM)	*k*_cat_ (s^−1^)	*k*_cat_/*K*_m_ (s^−1^ mM^−1^)
*p*NP-β-**d**-glucopyranoside	CbgA	1.77 ± 0.22	5.13 ± 0.19	2.90
	CbgB	1.13 ± 0.21	1.28 ± 0.066	1.13
*p*NP-β-**d**-galactopyranoside	CbgA	5.16 ± 0.24	29.5 ± 0.57	5.72
	CbgB	6.04 ± 0.70	8.19 ± 0.52	1.36
*p*NP-β-**d**-fucopyranoside	CbgA	0.191 ± 0.073	8.19 ± 0.49	42.8
	CbgB	0.0594 ± 0.021	2.05 ± 0.06	34.5

Collectively, these kinetic results indicate that CbgA is the dominant intracellular β-glucosidase, exhibiting higher catalytic efficiency toward the representative β-glycosides tested under our assay conditions, whereas CbgB displayed lower catalytic efficiency.

The optimal pH and temperature for CbgA and CbgB were determined using *p*NP-β-**d**-glucopyranoside as the substrate (Supplementary Fig. S3A and B). CbgA exhibited maximal activity at pH 6.0 (1.03 μmol/min/mg), whereas CbgB displayed maximal activity at pH 6.5 (0.813 μmol/min/mg). Both enzymes retained > 50% of their maximal activity within a relatively broad pH range (5.0–7.5; Supplementary Fig. S3A). Regarding temperature dependence, the activity of CbgA peaked at 45 °C (4.88 μmol/min/mg), whereas CbgB activity was optimized at 40 °C (2.57 μmol/min/mg; Supplementary Fig. S3B). Both enzymes displayed a sharp decline in activity at temperatures exceeding 50 °C, indicating that they are optimized for moderate temperature ranges rather than for high-temperature conditions.

### Substrate specificity of CbgA and CbgB

**Fig. 1:**
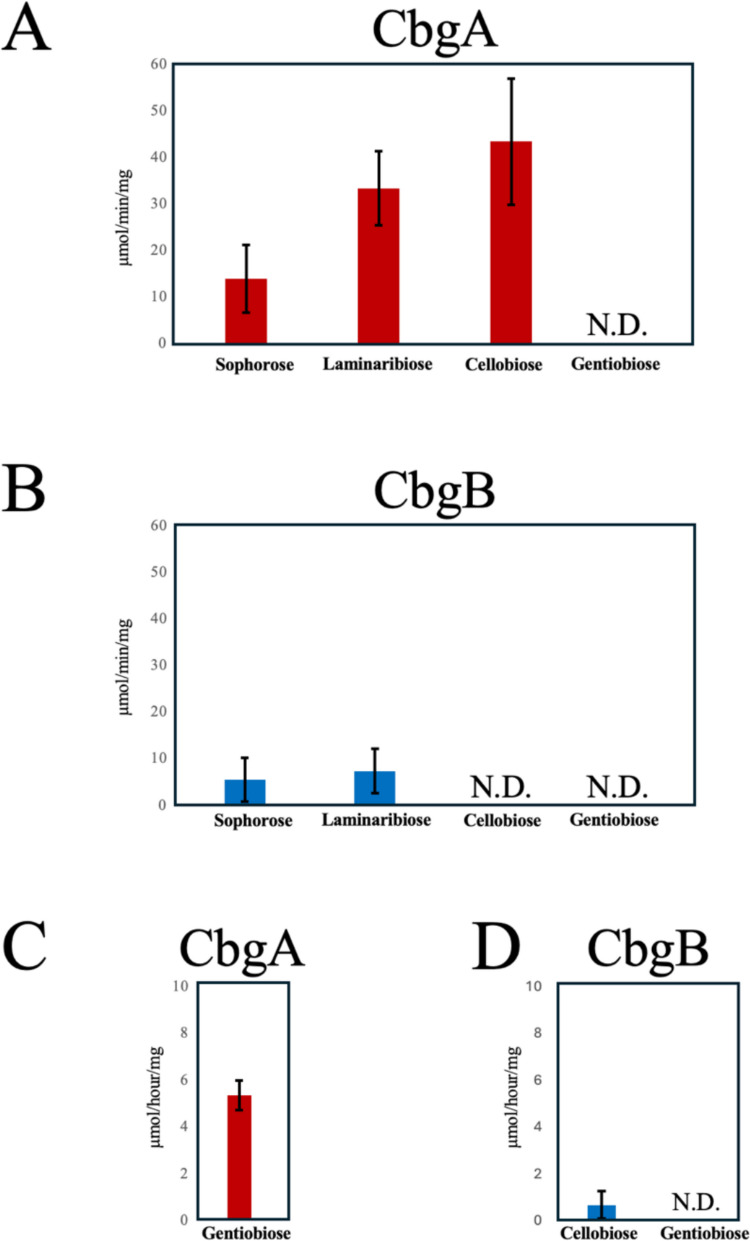
Substrate specificity of CbgA and CbgB toward β-linked disaccharides. **A**, **B** Hydrolytic activity of recombinant CbgA and CbgB toward cellobiose, laminaribiose, sophorose, and gentiobiose. Glucose release was measured after a 5-min reaction at 40 °C. **C**, **D** Time-course analysis of gentiobiose and cellobiose hydrolysis by CbgA (**C**) and CbgB (**D**) over 24 h. Data represent the mean ± s. d. (*n* = 3). N.D. indicates no detectable activity

We next evaluated the hydrolytic activities of CbgA and CbgB against cellobiose, laminaribiose, sophorose, and gentiobiose (Fig. 1A and B). Under a reaction time of 5 min, CbgA displayed the highest activity toward cellobiose (43.4 µmol/min/mg), followed by laminaribiose (33.4 µmol/min/mg) and sophorose (14.0 µmol/min/mg), whereas it exhibited no detectable activity toward gentiobiose under short-term reaction conditions. CbgB generally exhibited lower overall activities than CbgA, displaying the greatest activity toward laminaribiose (7.39 µmol/min/mg) and moderate activity toward sophorose (5.47 µmol/min/mg) but no detectable activity toward cellobiose and gentiobiose. Notably, when the reaction time was extended to 24 h, CbgA hydrolyzed gentiobiose (5.35 µmol/h/mg), indicating slow but detectable activity toward this substrate under prolonged incubation (Fig. 1C). Meanwhile, CbgB hydrolyzed cellobiose (0.67 µmol/h/mg) after 24 h but still lacked any measurable activity against gentiobiose (Fig. 1D). These results indicate that CbgA functions as a general intracellular β-glucosidase with broad substrate specificity, plays a major role in cellobiose degradation and contributes substantially to intracellular glucose release. Contrarily, CbgB plays a minor role in intracellular cellobiose degradation and lacks detectable ability to hydrolyze gentiobiose, even after prolonged incubation.

### Effects of glucose and organic solvents on enzyme activity

Product inhibition by glucose was evaluated by measuring residual activity in the presence of 0–1000 mM glucose (Supplementary Fig. S4A). The absolute specific activity in the absence of glucose was 2.07 μmol/min/mg for CbgA and 0.30 μmol/min/mg for CbgB. CbgA exhibited a concentration-dependent decrease in activity, retaining approximately 61% residual activity in the presence of 200 mM glucose but only 26% residual activity in the presence of 1000 mM glucose. CbgB displayed a markedly different response, as its activity increased to 134% in the presence of 400 mM glucose before gradually decreasing at higher concentrations; however, it retained approximately 80% of its activity when 1000 mM glucose was added. These findings demonstrate the high sensitivity of CbgA to glucose and the remarkable glucose tolerance of CbgB, highlighting a fundamental biochemical distinction between the two enzymes.

The stability of each enzyme in the presence of various organic solvents was also evaluated (Supplementary Fig. S4B). CbgA retained 76% of its activity in the presence of 10% (v/v) methanol, whereas CbgB only retained 59% of its activity under the same conditions. CbgB maintained 74% of its activity in the presence of 10% (v/v) ethanol, whereas CbgA activity declined substantially to 41% under this condition. In the presence of 5% (v/v) DMSO, CbgB retained 59% of its activity, whereas CbgA exhibited lower tolerance, retaining only 37% of its activity (Supplementary Fig. S4B). These results clearly demonstrate distinct solvent tolerance profiles between the two enzymes. These differences further support functional differentiation between the two enzymes at the biochemical level.

### Phenotypic analysis of *cbgA* and *cbgB* deletion mutants

**Fig. 2:**
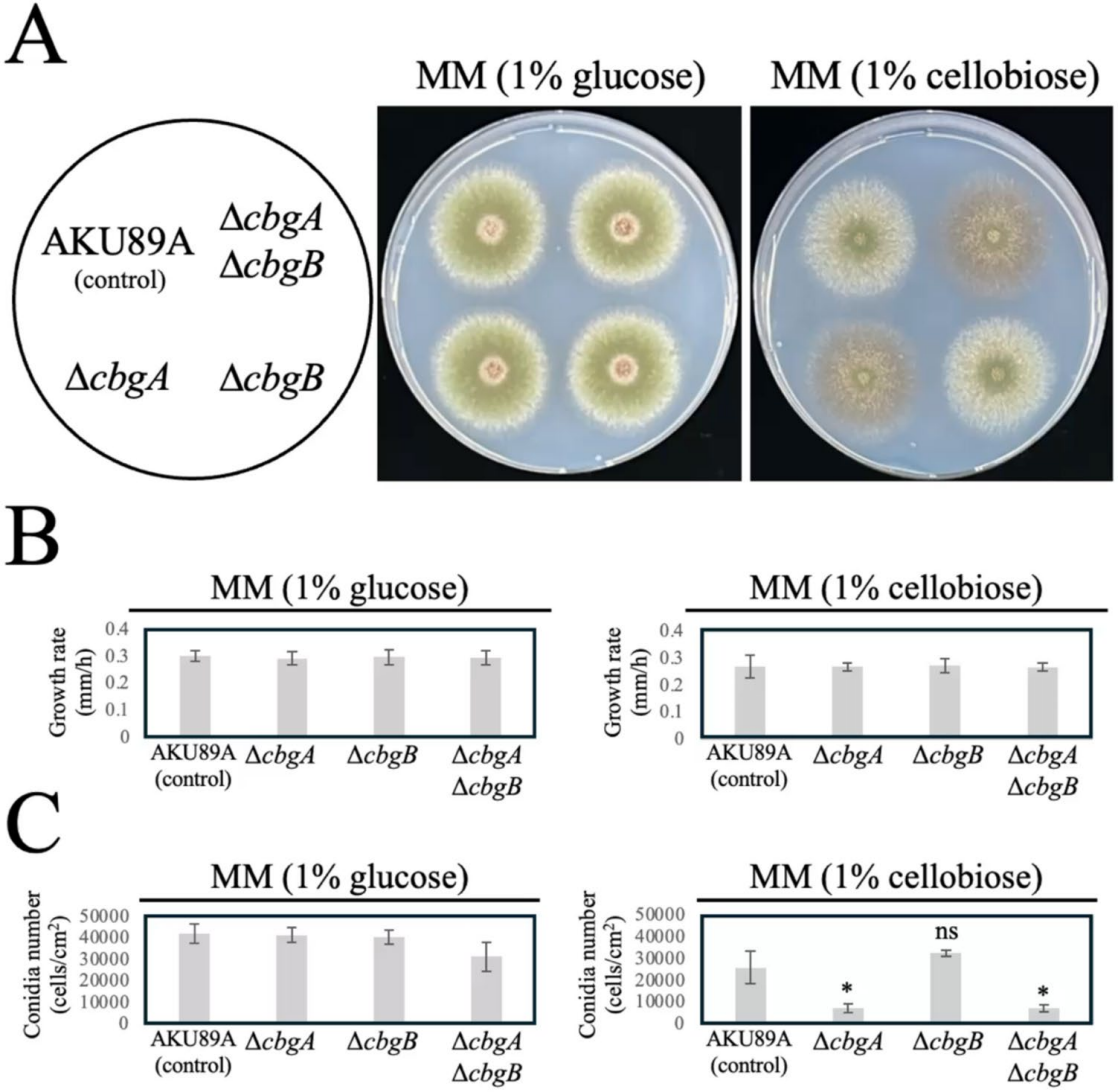
Phenotypic analysis of *cbgA* and *cbgB* deletion mutants. **A** Colony morphology of the parental (AKU89A), single-deletion (Δ*cbgA*, Δc*bgB*), and double-deletion (Δ*cbgA*Δ*cbgB*) strains. Conidial suspensions (1 × 10^4^ conidia) were spotted onto MM plates containing 1% glucose (left) or 1% cellobiose (right) and incubated at 37 °C for 3 days. **B** Hyphal extension rates on MM plates containing 1% glucose (left) or 1% cellobiose (right). Data represent the mean ± s. d. (*n* = 4). **C** Quantitative analysis of conidiation. Conidia were harvested from MM plates containing 1% glucose (left) or 1% cellobiose (right) after 3 days. The number of conidia was normalized to the parental strain. Bars represent the mean ± s. d. (*n* = 4). Statistical significance was evaluated by Welch’s *t*-test. All comparisons were performed against AKU89A. *p < 0.05; ns, not significant

Single-gene (Δ*cbgA*, Δ*cbgB*) and double-gene (Δ*cbgA*Δ*cbgB*) deletion mutants were constructed to investigate the physiological roles of CbgA and CbgB (Fig. 2). On MM (1% glucose) plates, all mutants exhibited similar colony morphology to the parental AKU89A strain, and conidiation levels were generally comparable, although the Δ*cbgA*Δ*cbgB* strain displayed a slight reduction in conidial formation (Fig. 2A). Furthermore, hyphal extension rates on MM (1% glucose) did not significantly differ among AKU89A, Δ*cbgA*, Δ*cbgB*, and Δ*cbgA*Δ*cbgB* (Fig. 2B). Conversely, striking differences were observed on MM (1% cellobiose) plates. Δ*cbgA* and Δ*cbgA*Δ*cbgB* colonies exhibited markedly reduced conidiation and developed a distinct reddish-brown pigmentation that was not observed in AKU89A or Δ*cbgB* (Fig. 2A). Quantitative analysis of conidiation (Fig. 2C) illustrated that although all strains produced comparable numbers of conidia on MM (1% glucose), Δ*cbgA* and Δ*cbgA*Δ*cbgB* formed significantly fewer conidia on MM (1% cellobiose), reaching only 27.8% and 27.3%, respectively, of the level of AKU89A. Statistical analysis using Welch’s *t*-test (*n* = 4) confirmed that these reductions were statistically significant between AKU89A and Δ*cbgA* (*p* = 0.0123) and between AKU89A and Δ*cbgA*Δ*cbgB* (*p* = 0.0128), whereas no statistical difference was recorded between Δ*cbgB* and the parental strain (*p* = 0.177).

To confirm that the observed phenotypes in the Δ*cbgA* strain resulted specifically from loss of function, a complementation strain was constructed by introducing a genomic fragment of the *cbgA* gene, including its native promoter and terminator regions, into the background. On MM plates containing 1% cellobiose, the strain exhibited similar conidiation levels and colony morphology as AKU89A (Supplementary Fig. S5). These results demonstrate that reintroducing the *cbgA* gene successfully rescued the cellobiose-dependent pigmentation and conidiation defects, confirming that these phenotypes are directly associated with the loss of CbgA activity. Taken together, these results indicate the critical role of CbgA, but not CbgB, in cellobiose metabolism, and that *cbgA* loss leads to impaired conidiation and increased reddish-brown pigment accumulation under cellobiose-utilizing conditions, potentially linking CbgA function to developmental regulation and secondary metabolism.

### Subcellular localization of CbgA and CbgB

**Fig. 3:**
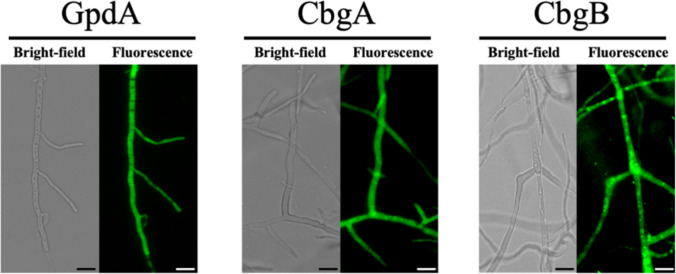
Subcellular localization of CbgA and CbgB in *A. nidulans*. Fluorescence microscopy images of strains expressing eGFP fusion proteins. GpdA-eGFP was used as a cytosolic control. Scale bars represent 10 μm.

To determine the intracellular localization of CbgA and CbgB, C-terminally eGFP-tagged fusion proteins were expressed in *A. nidulans* (Fig. 3). The cytosolic enzyme GpdA fused to eGFP served as a control (Punt et al. 1988). Fluorescence microscopy revealed that CbgA-eGFP displayed a similar diffuse cytosolic distribution to GpdA-eGFP, indicating that CbgA is predominantly localized in the cytosolic compartment (Fig. 3). Although CbgB-eGFP displayed a clear cytosolic distribution, punctate fluorescence patterns were also observed, suggesting that CbgB can also localize to vacuoles or other unidentified subcellular compartments (Fig. 3). These distinct localization patterns further indicate that CbgA and CbgB play different physiological roles in *A. nidulans*.

### Intracellular β-glucosidase activity in deletion mutants

**Fig. 4:**
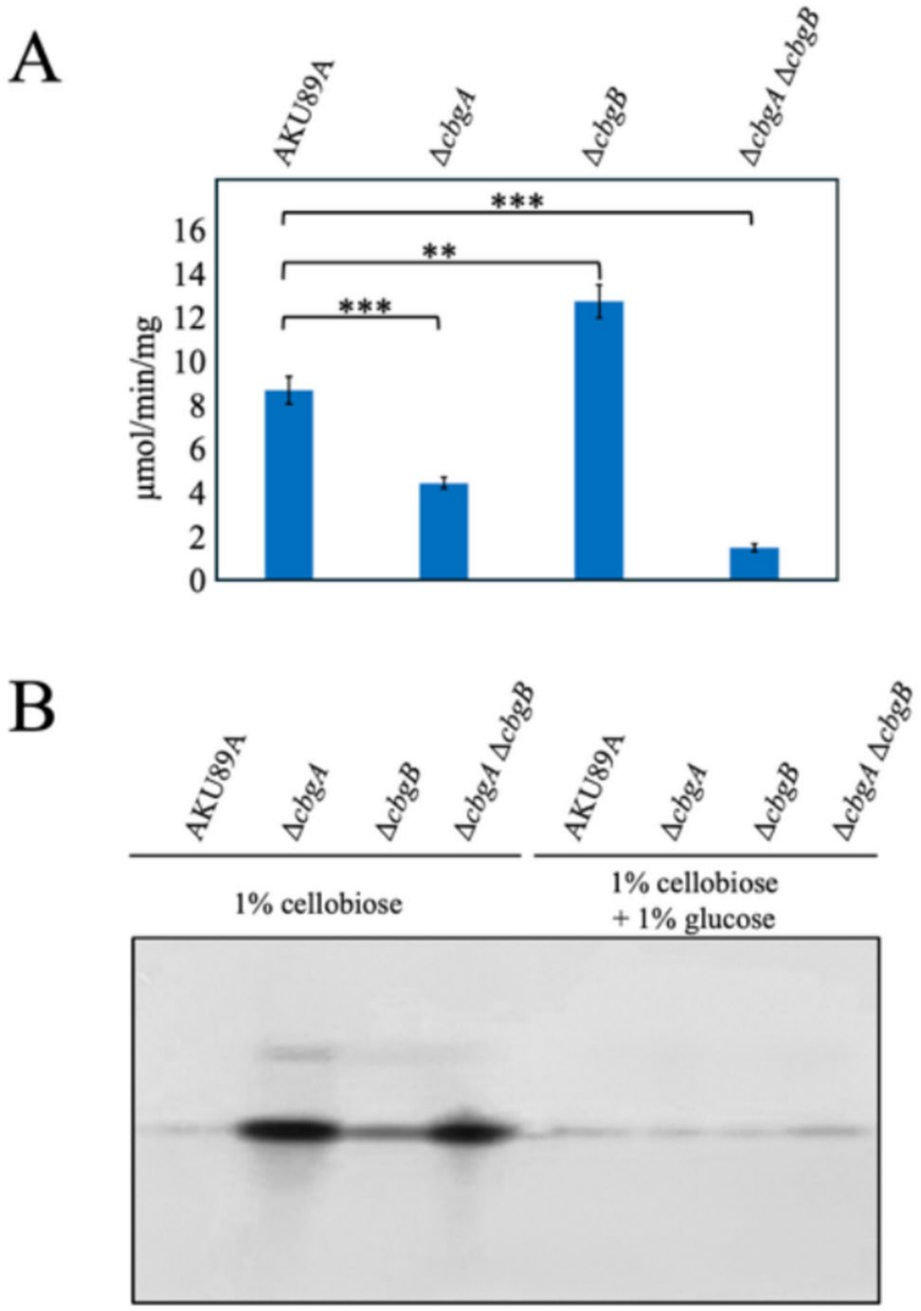
Intracellular β-glucosidase activity and extracellular cellulase activities. **A** Intracellular β-glucosidase activity in cytosolic fractions extracted from protoplasts. Activity was measured using *p*NP-β-**d**-glucopyranoside as the substrate. Bars represent the mean ± s.d. (*n* = 4). Statistical significance was evaluated by Welch’s *t*-test. All comparisons were performed against AKU89A. **p < 0.01; ***p < 0.001. **B** Congo red zymogram of cellulase activity in culture supernatants. Strains were grown on MM containing 1% cellobiose (left) or 1% cellobiose + 1% glucose (right) for 24 h

In *Aspergillus*, multiple β-glucosidases are associated with the cell wall (Lee et al. 1996; Iwashita et al. 1998, 1999), and simple mechanical homogenization of mycelia would release these enzymes into the extract, making it difficult to accurately assess the true intracellular β-glucosidase activity. To overcome this, protoplasts were first prepared by enzymatic cell wall removal, washed extensively, and then lysed to obtain a cytosolic fraction. β-Glucosidase activity in this cytosolic fraction was then quantified using *p*NP-β-**d**-glucopyranoside as the substrate (Fig. 4A). The Δ*cbgA* mutant exhibited substantially reduced intracellular β-glucosidase activity, decreasing to approximately 50% of the parental level (*p* < 0.001 by Welch’s *t*-test, *n* = 4), demonstrating that *cbgA* deletion alone is sufficient to cause a marked loss of intracellular activity. Furthermore, the Δ*cbgA*Δ*cbgB* strain only retained approximately 15% of the parental activity (*p* < 0.001), indicating that CbgA is the predominant intracellular β-glucosidase under these conditions (Fig. 4A). Interestingly, the Δ*cbgB* mutant displayed slightly higher activity than AKU89A (*p* < 0.01), suggesting a possible compensatory enhancement of CbgA expression or activity.

### Cellulase production in *cbgA* and *cbgB* deletion mutants

Cellulase secretion was evaluated using Congo red zymography of culture supernatants from *A. nidulans* strains grown in the presence of 1% cellobiose alone (left lanes in Fig. 4B) or in combination with 1% glucose (right lanes in Fig. 4B). In AKU89A, cellulase activity was barely detectable under either condition. Similarly, the Δ*cbgB* mutant displayed no significant cellulase induction in both cases. Conversely, the Δ*cbgA* and Δ*cbgA*Δ*cbgB* mutants exhibited markedly enhanced cellulase activity in the presence of cellobiose alone, as indicated by the strong activity bands observed in the zymogram. However, when glucose was added, cellulase induction was strongly suppressed in all strains, suggesting glucose-mediated catabolite repression (Kunitake and Kobayashi 2017). These results demonstrate that *cbgA* deletion leads to enhanced cellulase production, consistent with its role in modulating intracellular cellobiose levels and cellulase gene expression. As cellobiose activates the cellulase-specific transcription factor ClrB in *A. nidulans* (Znameroski et al. 2012; Yamakawa et al. 2013; Li et al. 2016; Kunitake and Kobayashi 2017), the enhanced cellulase production observed in the Δ*cbgA* and Δ*cbgA*Δ*cbgB* mutants can be attributed to increased intracellular cellobiose levels, leading to stronger ClrB activation. Conversely, CbgB appears to have little effect on cellulase regulation under the tested conditions.

### Cellobiose transporter CltB is required for Δ*cbgA*-associated pigmentation and cellulase induction

**Fig. 5:**
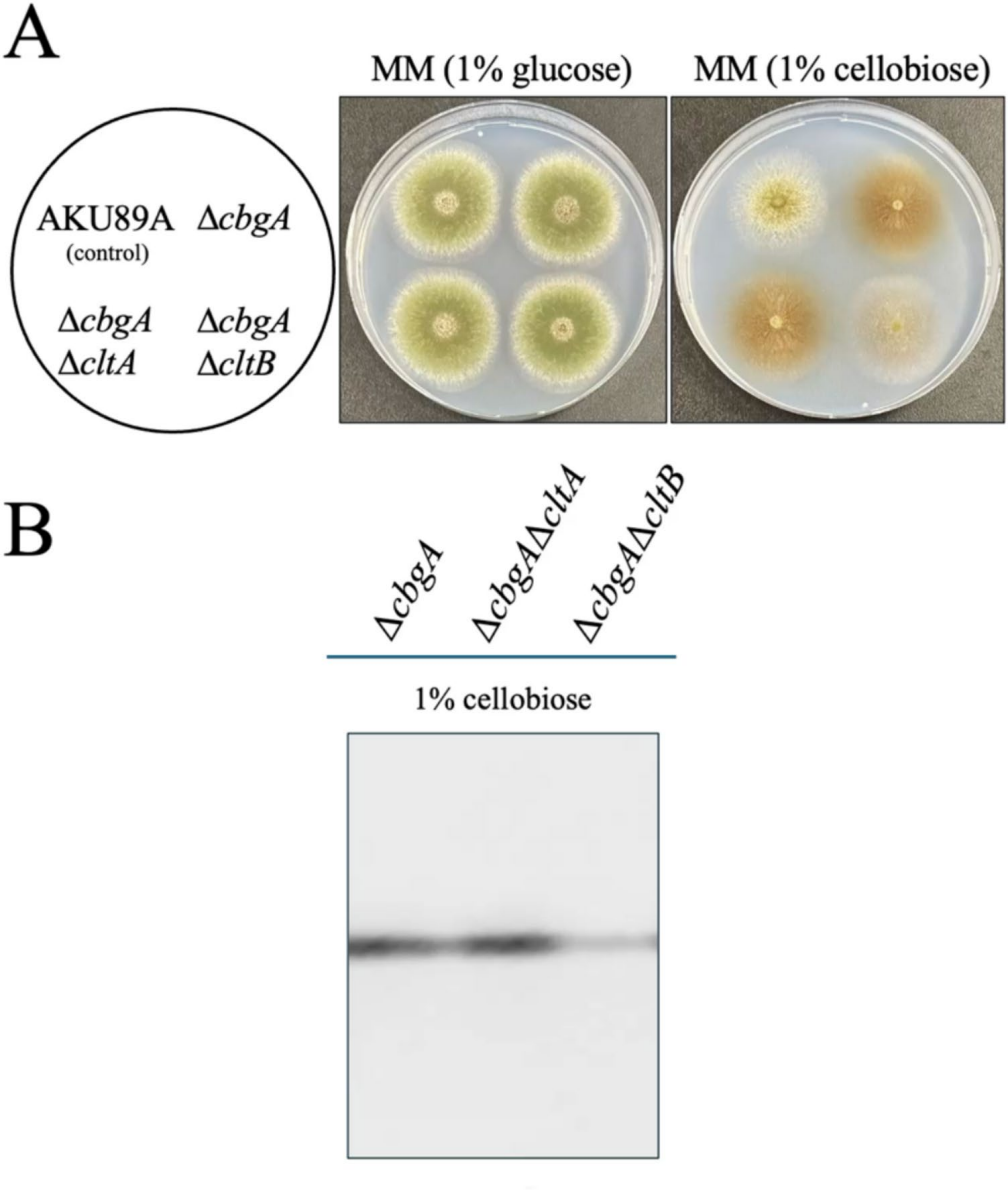
Requirement of cellobiose transporter CltB for Δ*cbgA*-associated phenotypes. **A** Colony morphology of Δ*cbgA*, Δ*cbgA*Δ*cltA*, and Δ*cbgA*Δ*cltB* after 3 days of growth on MM containing 1% glucose (left) or 1% cellobiose (right). **B** Congo red zymogram of cellulase activity. Strains were grown on MM containing 1% cellobiose for 24 h

To determine whether the Δ*cbgA*-associated phenotypes require intracellular cellobiose entry, we constructed double mutants lacking both *cbgA* and either of the cellobiose transporters *cltA* or *cltB* (Δ*cbgA*Δ*cltA* and Δc*bgA*Δ*cltB*) (Dos Reis et al. 2016). Because *cltB* is a major cellobiose transporter in *A. nidulans*, *cltB* deletion is expected to substantially reduce cellobiose uptake, thereby preventing intracellular cellobiose-dependent responses that would otherwise be modulated by CbgA (Dos Reis et al. 2016). In the presence of 1% glucose, all strains exhibited similar colony morphology (Fig. 5A). In the presence of 1% cellobiose, Δ*cbgA* produced the characteristic brown pigment, and Δ*cbgA*Δ*cltA* displayed a comparable pigmentation phenotype (Fig. 5A). Meanwhile, a clear reduction in brown pigment accumulation was observed in Δ*cbgA*Δ*cltB* on cellobiose (Fig. 5A). Congo red zymography under 1% cellobiose highlighted strong cellulase activity in *ΔcbgA* and Δ*cbgA*Δ*cltA*, whereas cellulase activity was reduced in Δ*cbgA*Δ*cltB* (Fig. 5B). Together, these results indicate that the pigmentation and cellulase induction observed in the Δ*cbgA* strain depend on cellobiose uptake via CltB.

## Discussion

### Functional differentiation of intracellular GH1 β-glucosidases

In this study, we characterized the intracellular GH1 β-glucosidases CbgA and CbgB in *A. nidulans*. Despite their significant sequence identities, our biochemical and genetic data revealed a clear functional role for CbgA as the primary intracellular enzyme. CbgA exhibited higher catalytic efficiency toward cellobiose and played a dominant role in maintaining total intracellular β-glucosidase activity. In contrast, CbgB displayed remarkable glucose tolerance and stability in organic solvents, suggesting that although it might not be the major enzyme for primary cellobiose catabolism, it could provide metabolic robustness under high-sugar or stressed conditions. This feature has been characterized in certain specialized GH1 enzymes, distinguishing them from typical glucose-sensitive β-glucosidases (Singh et al. 2016; Erkanli et al. 2024).

### CbgA as a negative regulator of cellulase induction

**Fig. 6:**
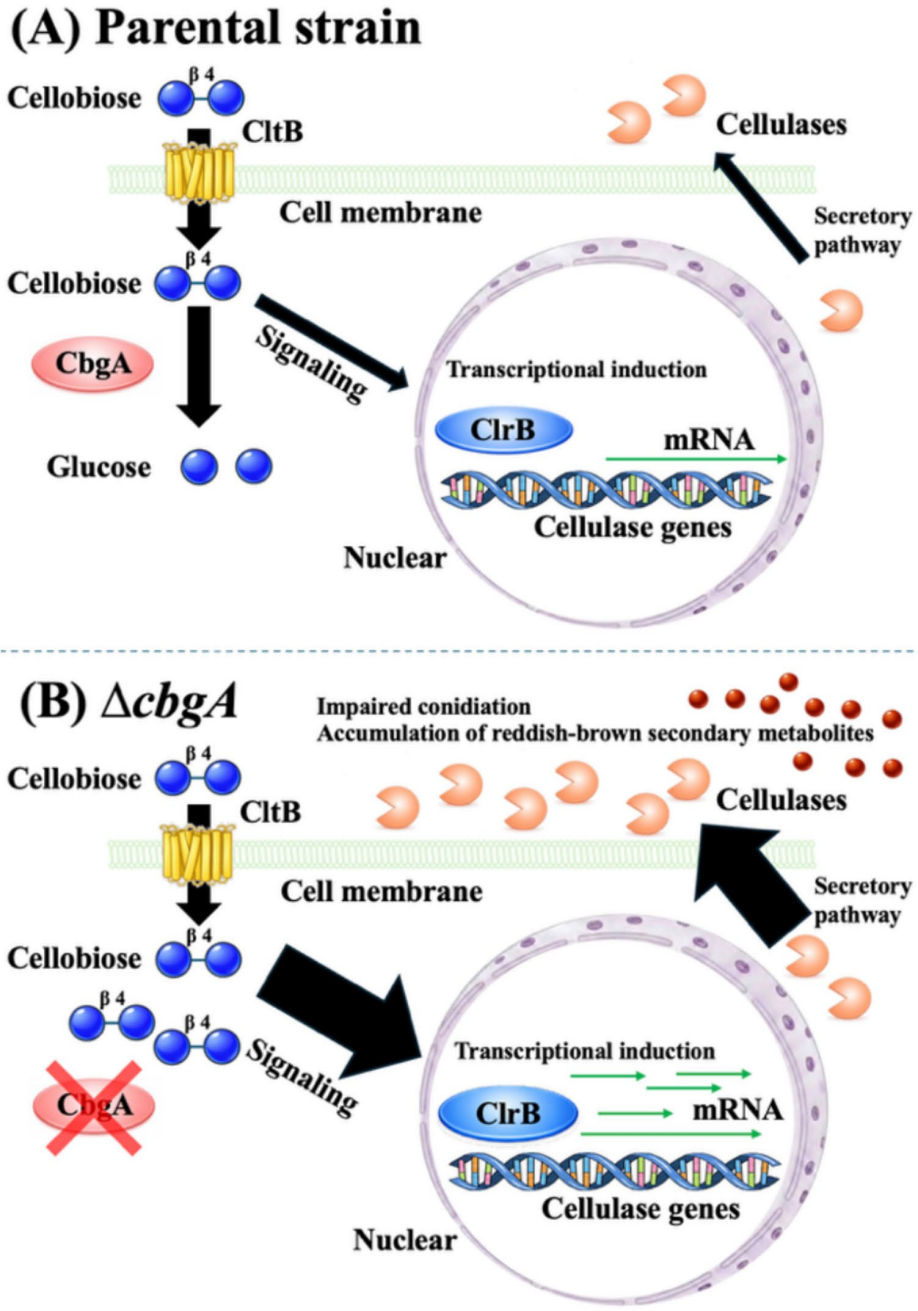
Proposed model for the role of CbgA in cellobiose-dependent signaling and development in *A. nidulans*. **A** In the parental strain, intracellular cellobiose is efficiently hydrolyzed by CbgA, restraining the induction signal within a physiological range for balanced cellulase expression and normal conidiation. **B** *cbgA* loss causes accumulation of intracellular cellobiose, leading to cellulase overactivation. The resulting secretory and metabolic stress leads to the accumulation of reddish-brown secondary metabolites and a decrease in conidiation

A central finding in this study was that CbgA negatively regulates cellulase expression. The Δ*cbgA* strain exhibited significantly enhanced cellulase activity on cellobiose (Fig. 4B). This is consistent with a model in which CbgA attenuates the cellulase-inducing signal by rapidly hydrolyzing intracellular cellobiose (Fig. 6). In *A. nidulans*, cellobiose activates the transcription factor ClrB (Chikamatsu et al. 1999; Kunitake and Kobayashi 2017). By reducing available inducer levels, CbgA prevents overactivation of the cellulolytic machinery. The significant enhancement of cellulase activity in the mutant indicates that CbgA primarily functions as a key negative regulator of the induction signal, rather than a mere nutrient provider. By regulating the induction signal, CbgA ensures that the metabolic response remains within a manageable range, a role that appears more critical for cellular fitness than glucose generation for energy. Similar regulatory roles for intracellular β-glucosidases have been proposed in other filamentous fungi. For instance, in *Penicillium decumbens*, restraining intracellular β-glucosidase activity promoted the production of extracellular lignocellulolytic enzymes (Chen et al. 2013). Consistently, recent genetic dissections in *T. reesei* and *A. niger* also indicated that intracellular β-glucosidases influence cellulase-related regulatory outcomes (Pang et al. 2021; Zhang et al. 2025). Together with these studies, our results support the view that intracellular β-glucosidases across various fungal genera can attenuate cellulase induction by reducing the intracellular levels of inducing oligosaccharides.

### Causal link between cellobiose uptake and Δ*cbgA* phenotypes

The most critical evidence supporting our model was the epistasis observed in Δ*cbgA*Δ*cltB*. The phenotypes of the Δ*cbgA* strain, namely enhanced cellulase induction, reddish-brown pigmentation, and impaired conidiation, were all significantly attenuated by additional deletion of the cellobiose transporter gene *cltB*. If the defects in the Δ*cbgA* strain were solely attributable to energy deficiency caused by a lack of glucose, further restriction of sugar uptake by *cltB* deletion would be expected to severely exacerbate growth impairment and the conidiation defect. Instead, the rescue of conidiation and reduction in pigmentation demonstrate that these defects are directly triggered by the intracellular accumulation of cellobiose or its related metabolic signaling. To explain how the ΔcbgA strain avoids complete starvation on cellobiose, it is important to note that *A. nidulans* produces several putative extracellular β-glucosidases, such as AN9183 (GH1), AN0712 (GH3), and AN4102 (GH3) (Saykhedkar et al. 2012). Previous studies found that extracellular β-glucosidase activity in this fungus is regulated by carbon catabolite repression (CCR) mediated by CreA (Lee et al. 1996). The presence of these extracellular enzymes suggests that a baseline glucose level might remain available to the Δ*cbgA* strain through the hydrolysis of cellobiose outside the cell. Therefore, the physiological role of CbgA more likely involves maintaining the homeostasis of the intracellular cellobiose pool than maximizing carbon catabolism for energy production. Although the *A. nidulans* genome encodes several secreted β-glucosidases, none can compensate for *cbgA* loss. This highlights that the cytosolic localization of CbgA is uniquely essential for its function as a signal gatekeeper. Although secreted enzymes in the GH1 and GH3 families contribute to extracellular glucose production, they cannot effectively hydrolyze the intracellular cellobiose pool that triggers the ClrB-mediated overactivation response. Furthermore, our data demonstrate that this intracellular induction signal remains under the strict control of global CCR. As shown in our zymogram analysis (Fig. 4B), the addition of glucose fully abolished cellulase secretion even in the ∆*cbgA* strain, confirming that CbgA-mediated signal attenuation does not bypass the primary CreA-dependent repression mechanism. Instead, CbgA regulation constitutes a distinct, second layer of control that fine-tunes the intensity of the induction signal specifically under inducing conditions. Ultimately, the intensity of the cellular response is determined by the balance between the rate of cellobiose entry via CltB and the speed of its subsequent hydrolysis by CbgA.

### Trade-off between cellulase overactivation and fungal development

The reduced conidiation in the Δ*cbgA* strain in the presence of cellobiose suggests a physiological trade-off between enhanced cellulase induction and fungal development. Notably, in filamentous fungi, nutrient limitation or starvation typically triggers asexual development as a survival strategy to disperse conidia (Skromne et al. 1995; Adams et al. 1998). However, the Δ*cbgA* strain exhibited the opposite response. Although *cbgA* loss might be expected to reduce glucose levels, conidiation was markedly impaired rather than promoted. This paradox indicates that the developmental defect is not the result of energy deficiency or glucose starvation. Instead, it suggests that the cell is experiencing overactivation stress. Although we did not experimentally assess unfolded protein response (UPR) activation in the Δ*cbgA* strain in the present study, the overactivation of cellulase genes can lead to severe endoplasmic reticulum (ER) stress because of the high demand on the protein-folding and secretion machineries. This secretory overload is known to trigger the UPR in several *Aspergillus* species (Sims et al. 2005; Guillemette et al. 2007; Zubieta et al. 2018; Tanaka et al. 2023). Our data suggest that CbgA protects cells from such metabolic or secretory overload by restraining the induction signal within a physiological range. The restoration of normal conidiation in the Δ*cbgA*Δ*cltB* double mutant further supports the notion that developmental inhibition is a secondary consequence of excessive signaling rather than a direct metabolic defect.

### Pigmentation and secondary metabolism

The cellobiose-dependent accumulation of reddish-brown pigment in the Δ*cbgA* mutant indicates crosstalk between carbon source signaling and secondary metabolism. Although the exact identity of this pigment remains to be determined, its abnormal accumulation suggests that impaired intracellular cellobiose degradation perturbs the normal regulation of secondary metabolism. These observations imply that CbgA contributes to maintaining proper pigmentation and secondary metabolism by ensuring optimal cellobiose metabolism.

### Future perspectives and study limitations

Despite these insights, the precise molecular mechanisms of cellulase induction remain to be fully elucidated. While it is well-established that cellobiose serves as the primary inducer in *A. nidulans*, our results highlight the critical role of the intracellular cellobiose pool as a major determinant of the induction response. The most significant remaining “black box” is the specific signaling cascade that bridges this intracellular pool with the activation of the transcription factor ClrB. Furthermore, while the correlation between cellobiose levels and altered secondary metabolism is evident, the specific chemical architecture and biosynthetic origin of the reddish-brown pigment warrant additional investigation. Future research utilizing comprehensive metabolomic and transcriptomic profiling will be essential to fully illuminate the regulatory networks governed by intracellular cellobiose homeostasis.

## Conclusion

In conclusion, CbgA is the major intracellular β-glucosidase in *A. nidulans*, and it plays a key role in modulating cellobiose-dependent signaling. By controlling the intracellular availability of cellobiose, CbgA prevents excessive cellulase production and protects normal developmental processes. From an industrial perspective, the targeted deletion or downregulation of *cbgA* homologs represents a potential strategy for enhancing cellulase titers in fungal cell factories, provided that the associated growth and secretion stresses are managed.

## Supplementary Information

Below is the link to the electronic supplementary material.

ESM 1 Supplementary Material 1 (PDF 2.25 MB)

## Data Availability

The datasets used during the current study are available from the corresponding author on reasonable request.
