# Supplementary material for: Intracellular β-glucosidase regulates cellulase expression and development in Aspergillus nidulans
Source: Appl Microbiol Biotechnol. 2026 May 4;110:187. doi: 10.1007/s00253-026-13851-9 (PMC13287295; doi:10.1007/s00253-026-13851-9)
Supplement: Supplementary file 1 [file 253_2026_13851_MOESM1_ESM.pdf]

Supplementary Material

Applied Microbiology and Biotechnology

Intracellular β-glucosidase regulates cellulase expression and development in *Aspergillus nidulans*

Shun Yakabe[1]†, Chihiro Kadooka[1, 2]†, Tomohiko Matsuzawa[3], Yuzuki Kawai[3], Masayuki Noguchi[2], Daisuke Hira[2], Masatoshi Goto[4], and Takuji Oka[1, 2]*

[1]Division of Applied Microbial Technology, Graduate School of Engineering, Sojo University, 4-22-1 Ikeda, Nishi-ku, Kumamoto, 860-0082, Japan.

[2]Department of Biotechnology and Life Sciences, Faculty of Biotechnology and Life Sciences, Sojo University, 4-22-1 Ikeda, Nishi-ku, Kumamoto, 860-0082, Japan

[3]Department of Applied Biological Science, Faculty of Agriculture, Kagawa University, 2393 Ikenobe, Miki, Kagawa, 761-0795, Japan

[4]Faculty of Agriculture, Saga University, 1 Honjo, Saga 840-8502, Japan.

†These authors contributed equally to this work.

*Corresponding author: oka@bio.sojo-u.ac.jp

Supplementary Table S1. Strains used in this study

Strains	Genotype
Aspergillus nidulans	
AKU89	*biA1*, Δ*nkuB::aurA*$^{+}$*, argB2*
AKU89P	*biA1*, Δ*nkuB::aurA*$^{+}$*, argB2*, Δ*pyrG*
AKU89A	*biA1*, Δ*nkuB::aurA*$^{+}$*, argB2::argB*
Δ*cbgA*	*biA1*, Δ*nkuB::aurA*$^{+}$*, argB2*, *cbgA::argB*
Δ*cbgB*	*biA1*, Δ*nkuB::aurA*$^{+}$*, argB2*, *cbgB::argB*
Δ*cbgA*Δ*cbgB*	*biA1*, Δ*nkuB::aurA*$^{+}$*, argB2*, Δ*pyrG, cbgA::argB, cbgB::pyrG*
Δ*cbgA*Δ*cltA*	*biA1*, Δ*nkuB::aurA*$^{+}$*, argB*, Δ*pyrG, cbgA::argB, cltA::pyrG*
Δ*cbgA*Δ*cltB*	*biA1*, Δ*nkuB::aurA*$^{+}$*, argB*, Δ*pyrG, cbgA::argB, cltB::pyrG*
AKU89A+pPTR-II-GpdA-eGFP	AKU89A harboring pPTR-II-GpdA-eGFP
AKU89A+pPTR-II-CbgA-eGFP	AKU89A harboring pPTR-II-CbgA-eGFP
AKU89A+pPTR-II-CbgB-eGFP	AKU89A harboring pPTR-II-CbgB-eGFP
AKU89A+ pPTR-II	AKU89A harboring pPTR-II
Δ*cbgA*+pPTR-II	Δ*cbgA* harboring pPTR-II
Δ*cbgA*+pPTR-II-CbgA	Δ*cbgA* harboring pPTR-II-cbgA

Supplementary Table S2 Sequences of synthetic genes and oligonucleotides used in this study

Oligo nucleotide primers	Sequence (5’→3’)
pET15-SmaI-CbgB-F	CTCTTTCAGGGACCCATGGGGTCTGTGGACAGTCCGGTACTGCCGTCTGACTTCCTCTGGGGCTTTGCAACCGC<br>GTCCTACCAGATTGAAGGTGCCGTGGACGAAGATGGTCGTGGTCCCAGCATTTGGGATACGTTCTGCAAGAAA<br>CCTGGGAAAATCGCTGGAGGAGCGAATGGTGATGTTGCGTGTGACAGCTATCATCGTACGCATGAGGACATC<br>GATCTGCTGAAACAGTGCCAAGCAAAAGCGTATCGCTTCTCTATTAGCTGGTCACGCGTTATTCCGTTGGGTG<br>GACGCAACGATCCGATCAATGAGAAAGGCTTGCAGTTTTACGTGAAGTTTGTGGATGATCTGCTCGCAGCTGG<br>TATTACCCCATTAGTTACCCTGTTTCACTGGGATTTACCTGAGGAGCTGGACAAACGCTATGGTGGGTTGCTGA<br>ACAAAGAGGAATTTGTAGCCGATTATGCGAATTATGCGCGGATCATTTTCAATGCTCTGAGTCCGAAAGTGAA<br>ATATTGGATTACATTTAACGAACCGTGGTGTTCGAGTGTCCTGGGTTACAACGTAGGCCAATTTGCCCCTGGCC<br>GTACCTCGGATCGGTCCAAGAATCCGGAAGGCGATGGCTCAACCGAACCGTGGATTGTGGGTCACAACATCCT<br>TGTTGCACATGGCACGGCGGTGAAAATCTACCGCGAAGAATTCAAGGCTCGCGATGGTGGTGAAATCGGCATT<br>ACCCTCAATGGCGATTGGGCAGAACCGTGGGACCCCGAAAACCCTGCCGACGTTGAAGCGGCCCCACGTAAG<br>ATTGAATTCGCGATTAGCTGGTTTGCTGATCCGATCTACTTCGGCCGTTACCCGGAAAGCATGATCAAACAGCT<br>TGGAAATCGTTTACCGGAATGGACTCCGGAAGAAGTGGCACTGGTCAAAGGTAGCAATGACTTTTATGGCATG<br>AACCACTACTGCGCCAACTTCATTCGCGCGAAAACATCGGAACCAGATCCGACGGATGTCGCCGGCAATCTTG<br>AGCTGCTGCTGCAGAACAAAGCCGGCGAATGGGTTGGCCCGGAAACTCAGTCACCTTGGTTACGTCCAAGTCC<br>CACCGGCTTCCGTAAACTGCTGAAATGGTTGTCGGATCGCTATAATCGCCCCAAAATTTACGTGACCGAAAAC<br>GGGACATCTCTGAAAGGGGAAAACGATCTGCCACTGGAACAACTCCTGAAAGACGATTTTCGTGTCAAGTACT<br>TTGAGGATTATATCCATGCGATGGCAGAGGCGTATACGTATGACAATGTCAACGTACGCGCGTATATGGCCTG<br>GTCCCTTATGGATAACTTCGAATGGGCCGAAGGTTATGAGACTCGCTTTGGGGTGACCTATGTTGACTACGAG<br>AACAACCAAAAGCGCTATCCGAAAGCATCCGCTAAAGCGATGAGCGAGATCTTTGCCAAATACATTCAGAAT<br>GAATAAGGGTACCAGGATCCG
pET15-SmaI-CbgB-F	CTCTTTCAGGGACCCATGGACTTGACTTCAGTGCA
pET15-SmaI-CbgB-R	CGGATCCTGGTACCCCTATGAAGCAATCAAATGCTCA
(GGGGS)$_2$-eGFP-F	GGTGGCGGTGGCAGCGGC
(GGGGS)$_2$-eGFP-R	TCGAGCTCGGTACCCCGCGCAGTAGAGCATGGTTAT
AN10124-F	CTCTAGAGGATCCCCATGATGGAGCACCTGGCCAT
AN10124-R	GCTGCCACCGCCACCCTCATTCTGGATATACTTCGCA
AN10375-F	CTCTAGAGGATCCCCTCGAAAGGGCAGCTCCAAGA
AN10375-R	GCTGCCACCGCCACCTGAAGCAATCAAATGCTCAAAC
AN8041-F	CTCTAGAGGATCCCCGCTGATTCTGGAGTGACCCA
AN8041-R	GCTGCCACCGCCACCTTGGGCATCAACCTTGGAGA
pPTR-II-conf-R	GTAAAACGACGGCCAGTG
cbgB-FC	GTGGTTGTGTTGAGTTCCTAC
cbgB-RC	GGCAGCTAAAGGAATCTCAG
cbgB-1	CGGGCTGCAGGAATTGATTTCCTCGTTGATGATGTC
cbgB-2	AGAGTCGACCCCTCGGTACTGATCCCTAGTGCGTTG
cbgB-3	CCCATCGATGGGGTACTTCATAGTCGCCCAGAATG
cbgB-4	GCTTGATATCGAATTCTTCTCAGCTTGCTCAATG
cbgA-FC	GCTTCCATACCAGAATCTGG
cbgA-RC	CATCCTAGGCATGGGAATATC
cbgA-1	CGGGCTGCAGGAATTGGAGAAGAGCTAGATGAGACC
cbgA-2	AGAGTCGACCCCTCGCTACAGAGCCCATGGTATG
cbgA-3	CCCATCGATGGGGTAGAGTGAGATTGTCGCTTGTG
cbgA-4	GCTTGATATCGAATTCGCTTCCTCTCTGATCAAG
AN8347(CltA)-FC	TGGTAAGGACCCTTGTTGC
AN8347(CltA)-RC	GCAGGCTTGAGAAGAGGTAC
AN8347(CltA)-1	TAGATGCATACGTGCCCAG
AN8347(CltA)-2	AGAGTCGACCCCTCGAGTTCACTTTGGGCCACTC
AN8347(CltA)-3	CCCATCGATGGGGTAGCCATCTTCATCATTAAGAGTG
AN8347(CltA)-4	CGTTATTACGTTGCTCACATTC
AN2814(CltB)-FC	CAAGCCAAAGTCTCGCTTAG
AN2814(CltB)-RC	GGTGAAGCAACCTAAGGAGC
AN2814(CltB)-1	TTGCTAGTGATTCAGACCGG
AN2814(CltB)-2	AGAGTCGACCCCTCGTGAAGACAGACTTGGTGGTG
AN2814(CltB)-3	CCCATCGATGGGGTATACCCTCGAAGAACTCGAGT
AN2814(CltB)-4	CTGAAACTAAGCTGCTAGCACG
pHSG396-F	CGAGGGGTCGACTCTAGAGG
pHSG396-R	TACCCCATCGATGGGGGATC
pPTR-II-cbgA-F	CTCTAGAGGATCCCCGACCGCCAGCTATCTTATGA
pPTR-II-cbgA-R	TCGAGCTCGGTACCCGCACTTGGCTGCAATACATC

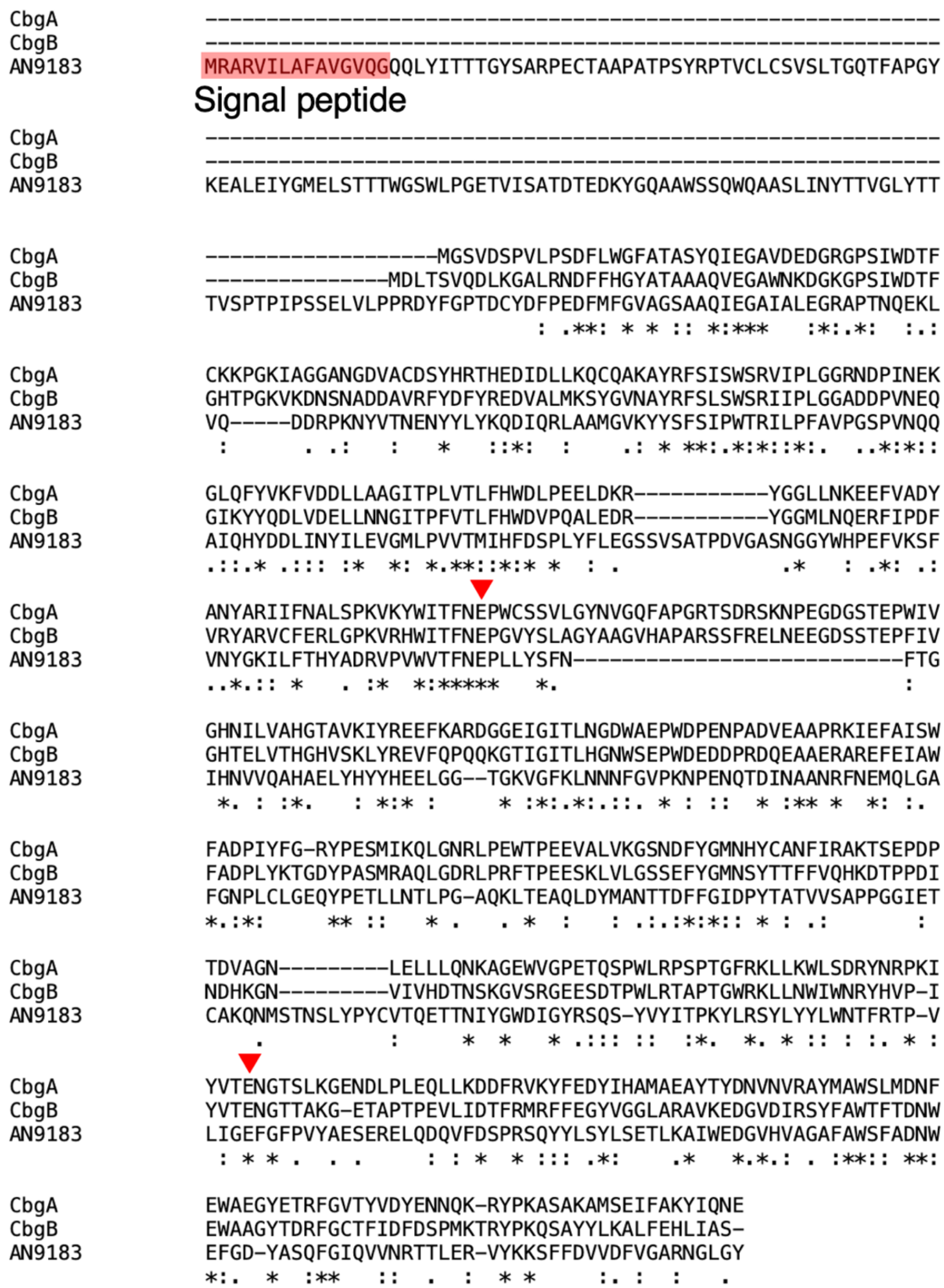

Supplementary Fig. S1. Multiple sequence alignment of *Aspergillus nidulans* GH1 β-glucosidases. The amino acid sequences of the cytosolic enzymes CbgA (AN10124) and CbgB (AN10375), and the predicted secretory enzyme AN9183, were aligned. AN10353, another GH1 family protein, was excluded from this alignment due to its extremely low sequence homology (< 10% identity) to CbgA and CbgB. The putative N-terminal signal peptide of AN9183, predicted by SignalP 6.0, is highlighted in red. The absence of this signal sequence in CbgA and CbgB is consistent with their predicted cytosolic localization. Identical (*), strongly conserved (:), and weakly conserved (.) residues are indicated below the alignment. The red arrows indicate the conserved acidic catalytic residues typical of the GH1 family.

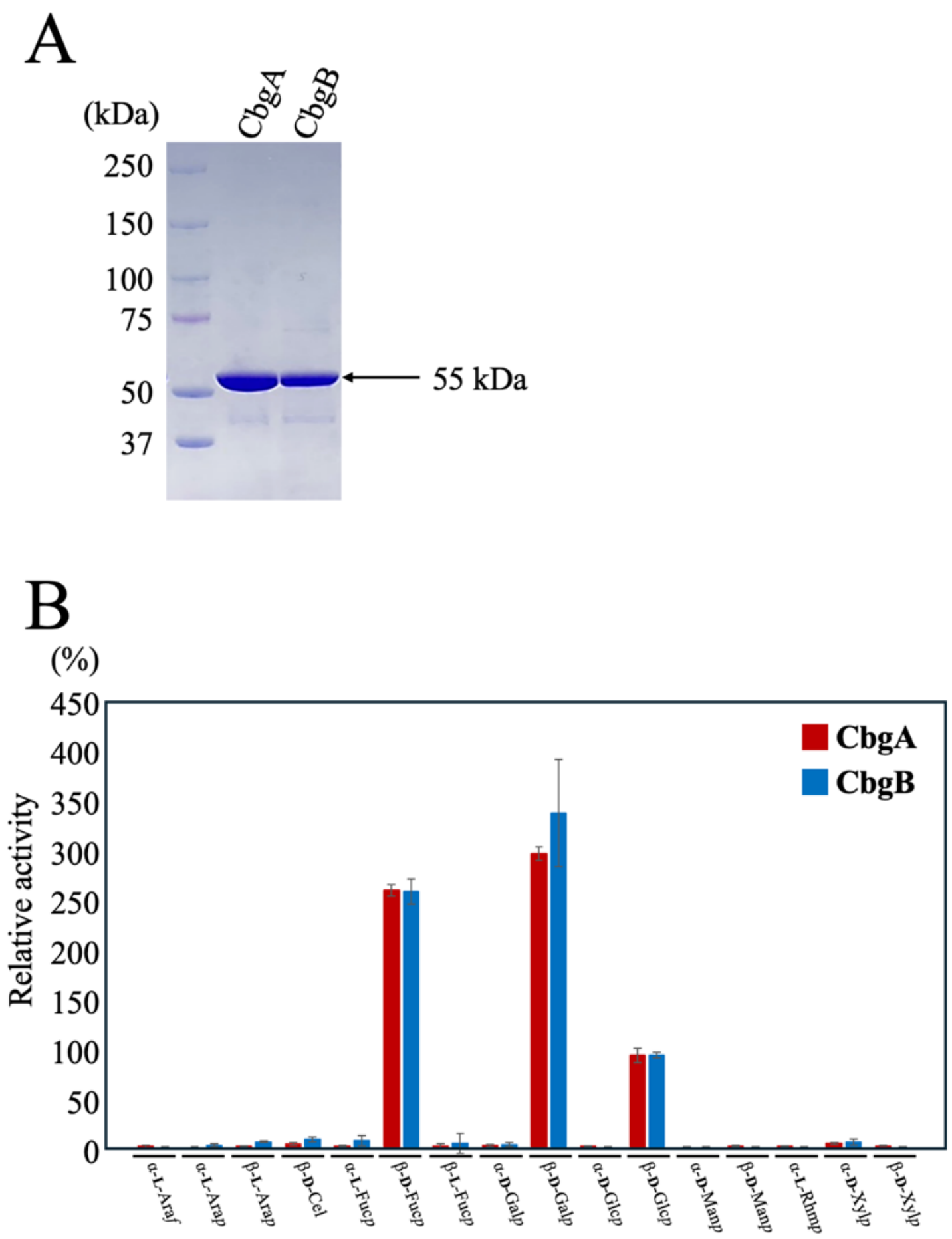

Supplementary Fig. S2. Purification and substrate screening of recombinant CbgA and CbgB. (A) SDS-PAGE analysis of purified recombinant CbgA and CbgB expressed in *E. coli*. Proteins were purified by Ni-NTA affinity chromatography and stained with Coomassie Brilliant Blue R-250. M: molecular weight markers. **(B)** Substrate specificity screening of CbgA and CbgB against 16 different *p*-nitrophenyl (*p*NP) glycosides. The reaction mixture (20 µL) containing 2 mM pNP substrate, 20 mM sodium phosphate buffer (pH 6.5), and 0.1 µg CbgA or CbgB was incubated at 40°C for 10 min. After the reaction, 100 µL of 1 M $NaHCO_3$ was added to stop the reaction. The concentration of the released pNP was measured at 405 nm. The relative activities were calculated by setting β-glucosidase activity to 100%. Data represent the mean ± s. d. (*n* = 3). The substrate abbreviations are defined as *p*NP-α-L-arabinofuranoside (α-L-Ara*f*), *p*NP-α-L-arabinopyranoside (α-L-Ara*p*), *p*NP-β-L-arabinopyranoside (β-L-Ara*p*), *p*NP-β-D-cellobioside (β-D-Cel), *p*NP-α-L-fucopyranoside (α-L-Fuc*p*), *p*NP-β-D-fucopyranoside (β-D-Fuc*p*), *p*NP-β-L-fucopyranoside (β-L-Fuc*p*), *p*NP-α-D-galactopyranoside (α-D-Gal*p*), *p*NP-β-D-galactopyranoside (β-D-Gal*p*), *p*NP-α-D-glucopyranoside (α-D-Glc*p*), *p*NP-β-D-glucopyranoside (β-D-Glc*p*), *p*NP-α-D-mannopyranoside (α-D-Man*p*), *p*NP-β-D-mannopyranoside (β-D-Man*p*), *p*NP-α-L-rhamnopyranoside (α-L-Rhm*p*), *p*NP-α-D-xylopyranoside (α-D-Xyl*p*), and *p*NP-β-D-xylopyranoside (β-D-Xyl*p*).

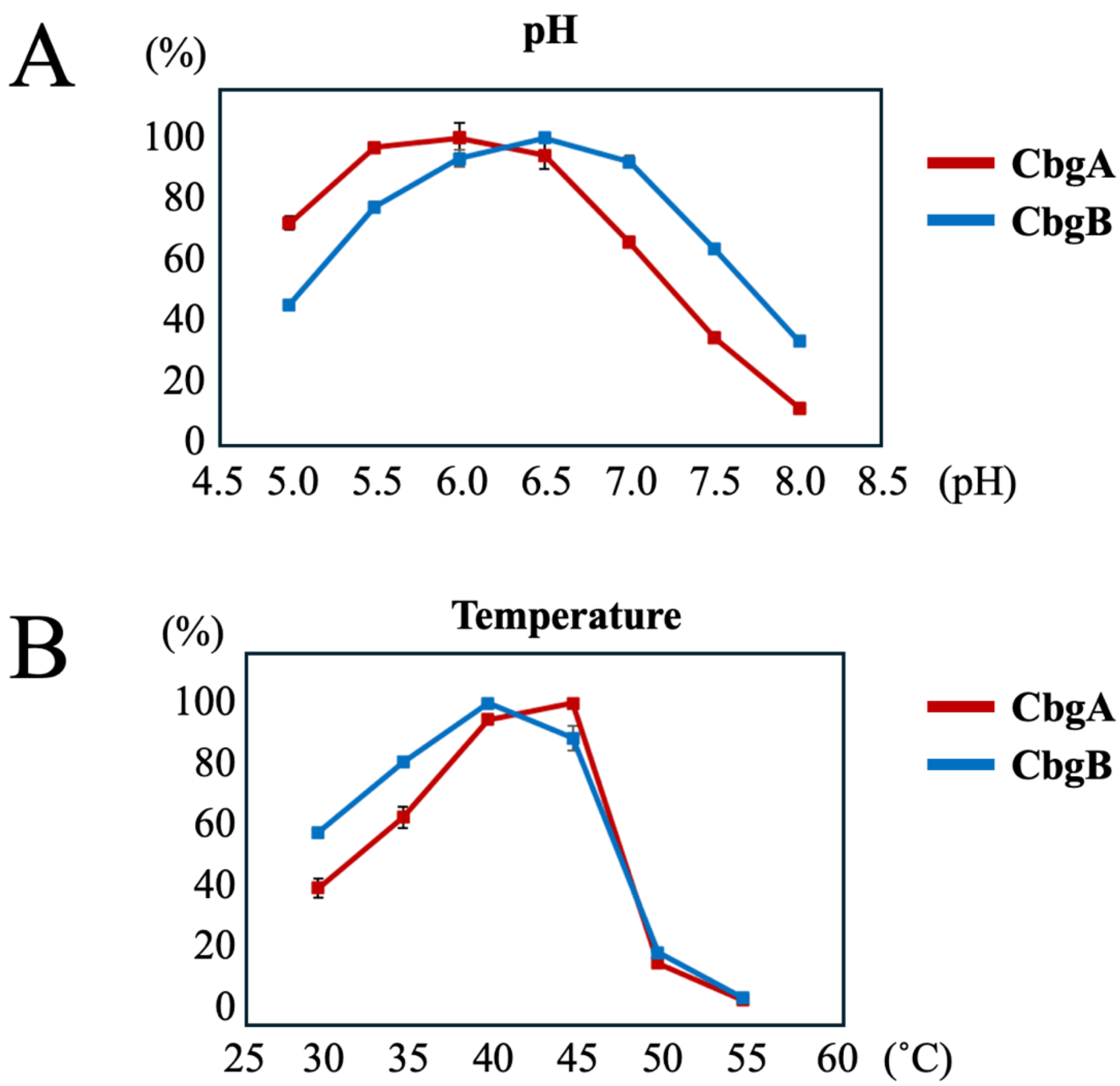

Supplementary Fig. S3. Effects of pH and temperature on CbgA and CbgB activity. (A) Optimal pH profiles of CbgA and CbgB. McIlvaine buffer (pH 5.0–8.0) was used. Activity was measured using *p*NP-D-glucopyranoside as the substrate across a pH range of 5.0 to 8.0. The maximum activity for each enzyme (at pH 6.0 for CbgA and pH 6.5 for CbgB) was defined as 100%. Data represent the mean ± s. d. (n = 3). **(B)** Optimal temperature profiles of CbgA and CbgB. Activity was measured at temperatures ranging from 30°C to 55°C. The maximum activity for each enzyme (at 45°C for CbgA and 40°C for CbgB) was defined as 100%. Data represent the mean ± s. d. (n = 3).

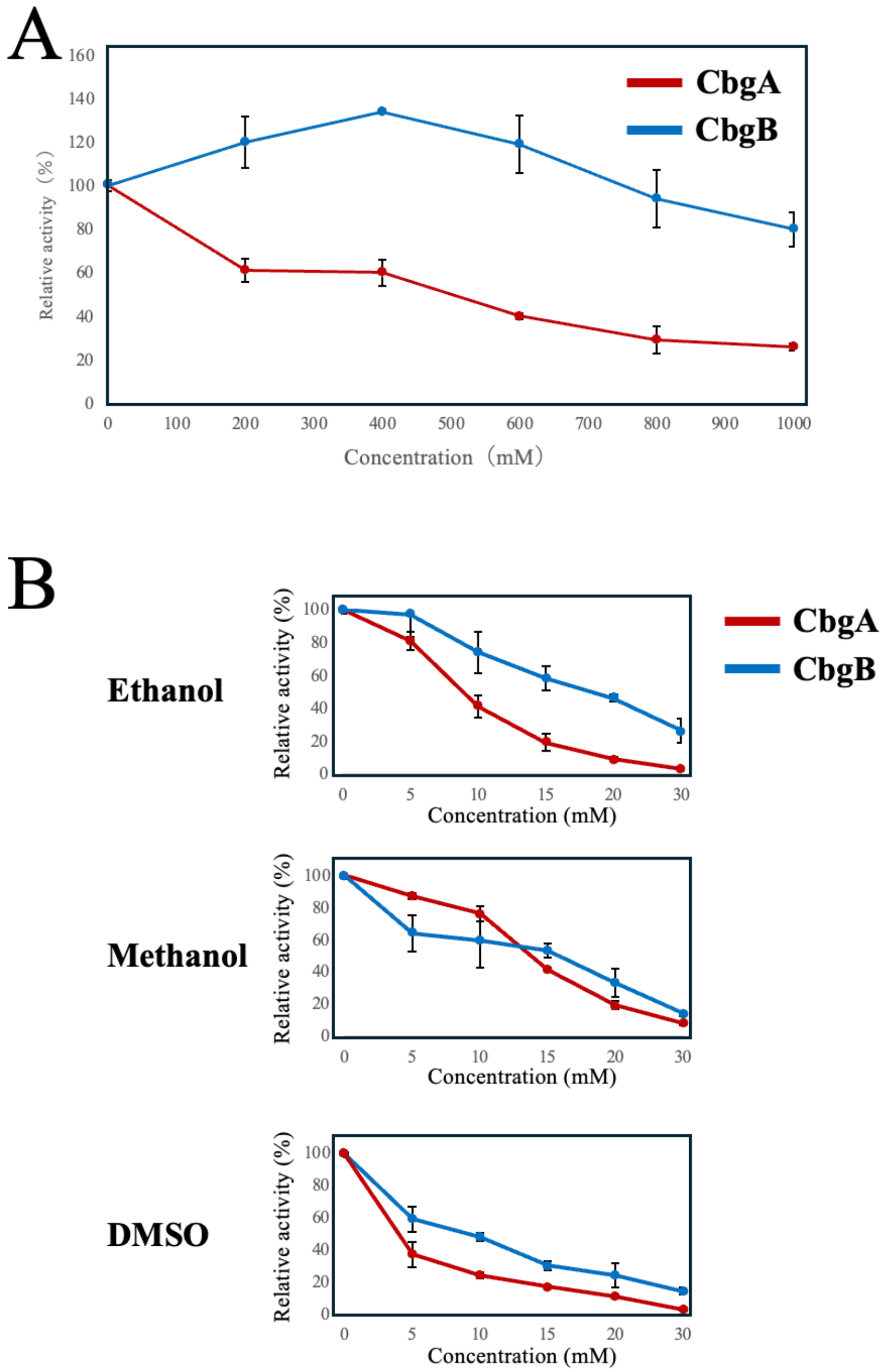

Supplementary Fig. S4. Effects of glucose and organic solvents on CbgA and CbgB activity. (A) Glucose tolerance of CbgA and CbgB. Residual activity was measured in the presence of 0 to 1000 mM glucose using *p*NP-D-glucopyranoside as the substrate. Data represent the mean ± s. d. (*n* = 3). **(B)** Solvent resistance of CbgA and CbgB. Residual activity was measured in the presence of various concentrations (0% to 30% v/v) of methanol, ethanol, or DMSO. Data represent the mean ± s. d. (*n* = 3).

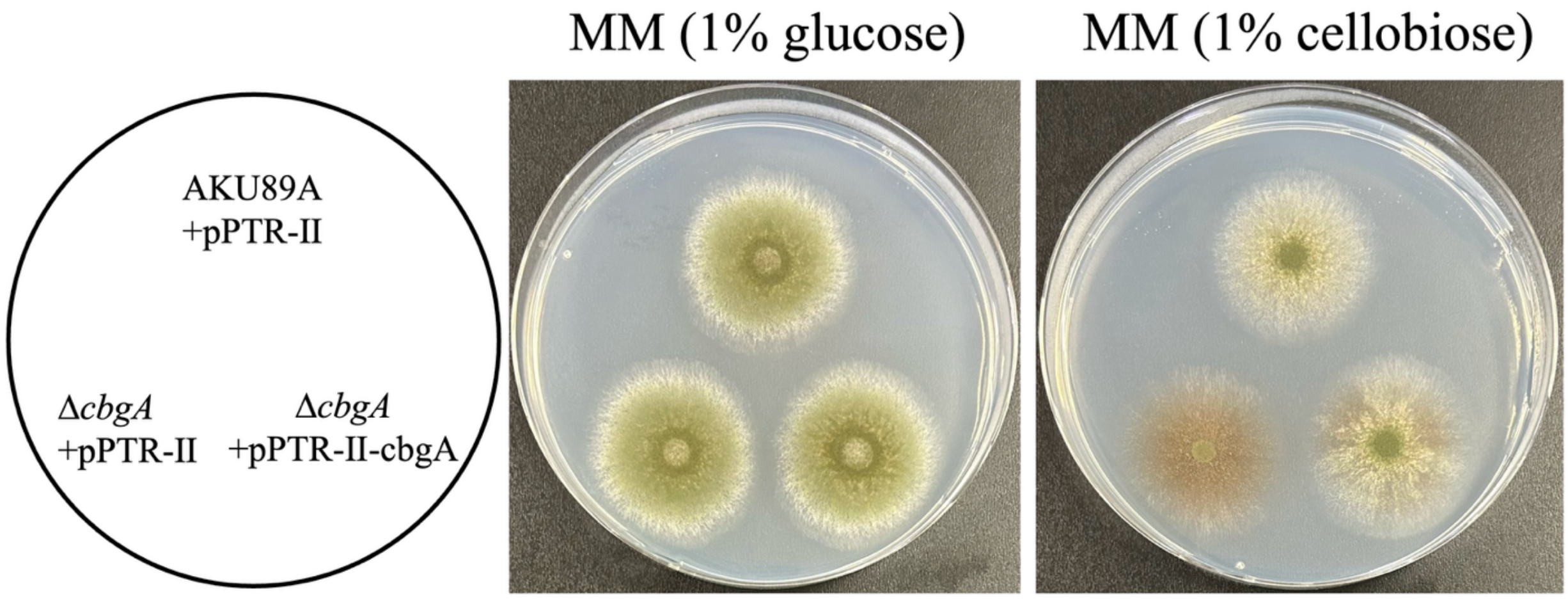

Supplementary Fig. S5. Phenotypic rescue of the mutant by gene complementation. Colony morphology and conidiation of the parental (AKU89A + pPTR-II), Δ*cbgA* (Δ*cbgA* + pPTR-II), and *cbgA* complementation strain (Δ*cbgA* + pPTR-II-cbgA). Conidial suspensions were spotted onto MM plates containing 1% glucose (*left*) or 1% cellobiose (*right*) and incubated at 37°C for 3 days. Reintroduction of the *cbgA* gene restored normal conidiation and markedly attenuated the reddish-brown pigmentation observed in the Δ*cbgA* strain.
